# Interactions Between Na⁺/K⁺-ATPase Inhibition and Protein Kinase C Signaling in Bipolar Disorder: Insights from Translational Data

**DOI:** 10.1007/s11064-026-04924-2

**Published:** 2026-10-22

**Authors:** Jorge M. Aguiar-Geraldo, Jefté Peper-Nascimento, Taise Possamai-Della, Thiani Daminelli, Tatiéli Scheffer-Luiz, Vitória Bisognin-Ceretta, Luciane B. Ceretta, João Quevedo, Samira S. Valvassori

**Affiliations:** 1 Laboratório de Psiquiatria Translacional, Programa de Pós-Graduação em Ciências da Saúde, Universidade do Extremo Sul Catarinense (UNESC), Criciúma, SC Brazil; 2 Programa de Pós-Graduação em Saúde Coletiva, Universidade do Extremo Sul Catarinense (UNESC), Criciúma, SC Brazil; 3 Center for Interventional Psychiatry, Faillace Department of Psychiatry and Behavioral Sciences, McGovern Medical School, The University of Texas Health Science Center at Houston (UTHealth Houston), Houston, TX USA

**Keywords:** Bipolar disorder, Na + K+ATPase, Protein kinase C, Oxidative stress

## Abstract

Protein Kinase C (PKC) dysregulation can affect Na⁺/K⁺-ATPase function, while alterations in Na⁺/K⁺-ATPase activity can conversely influence the PKC pathway. Therefore, this study investigated the interactions between Na⁺/K⁺-ATPase inhibition and PKC signaling, as well as the associated oxidative stress, using a translational approach that combines preclinical (in vitro and in vivo) models and clinical data from patients with bipolar disorder. In the in vitro model, cells were exposed to ouabain (OUA), a Na^+^/K^+^ ATPase inhibitor, and then treated with a PKC inhibitor (PKCi - tamoxifen). In the in vivo model, animals received intracerebroventricular OUA and were treated with PKCi. The animals underwent behavioral tests to evaluate manic- and depressive-like behaviors. The clinical study included a case-control protocol with 36 BD patients and 46 healthy controls. In both preclinical and clinical settings, the markers (4-hydroxynonenal [4-HNE], 8-isoprostane [8-ISO], thiobarbituric acid reactive substances [TBARS], carbonyl, and 3-nitrotyrosine [3-nitro]), as well as Na+/K+ ATPase and PKC activities, were assessed. In the in vivo and in vitro protocols, OUA increased PKC activity and reduced Na^+^/K^+^ ATPase activity. However, when combined with PKCi, OUA failed to inhibit Na^+^/K^+^ ATPase activity. Additionally, OUA was able to induce manic- and depressive-like behaviors and oxidative stress, which was also reversed by PKCi. Moreover, the clinical protocol identified increased PKC activity and reduced Na+/K+ ATPase activity, consistent with the preclinical protocols. Taken together, the data indicates that the interconnection between Na⁺/K⁺-ATPase and PKC signaling pathways represents an important component of BD pathophysiology.

## Introduction

Bipolar disorder (BD) is a major affective disorder characterized by severe mood swings, ranging from mania/hypomania to depressive episodes. BD has an estimated global lifetime prevalence of 0.6–1.6% [1]. From a neurobiological perspective, BD is complex and involves biological disturbances across several pathways, including imbalances in neurotransmitter systems, mitochondrial dysfunction, changes in immune-inflammatory systems, and oxidative stress [2, 3]. One theory that encompasses all these pathways is the alteration of sodium-potassium adenosine triphosphatase (Na^+^/K^+^ ATPase) [4]. Na^+^/K^+^ ATPase is a transmembrane enzyme responsible for maintaining electrolyte homeostasis in almost all eukaryotic cells. The brain, in particular, has high expression and demand for this enzyme, which is crucial for reestablishing action potentials in neurons and for neurotransmitter reuptake in astrocytes, for example [5].

In the context of BD, Na^+^/K^+^ ATPase activity appears to be decreased, as noted by many previous studies [6–8]. One of the causes of this alteration is endogenous cardiac steroids (ECS), mainly ouabain (OUA) [9, 10]. This group of molecules is elevated in patients with BD, compared to patients with schizophrenia, depression, and healthy controls [11]. However, it is important to note that some evidence suggests Na^+^/K^+^ ATPase could be hyperactivated and ECS levels reduced [12, 13]. Ultimately, when the activity of this enzyme is altered, either by inhibition or hyperactivation, it leads to changes in neuronal activity, oxidative stress, and cell growth, contributing to the symptomatology of BD [12]. Additionally, alterations in Na+/K+ ATPase function and ECS levels are closely intertwined with a complex network of intracellular signaling pathways, including Akt, ERK, MEK, and PKC [14].

The PKC family consists of 10 serine/threonine kinases, subdivided into 3 subfamilies based on their regulatory structure and cofactors: classical (cPKC), novel (nPKC), and atypical (aPKC) [15]. PKC signaling plays a crucial role in several pathological mechanisms, such as neuronal excitability, neurotransmitter release, and long-term alterations in gene expression and plasticity [16]. Interestingly, evidence suggests a complex interaction between these systems; for instance, Na+/K+ ATPase inhibition by OUA has been associated with PKC activation under specific cellular conditions [17]. Conversely, PKC signaling is reported to modulate Na+/K+ ATPase activity and trafficking, potentially influencing its cell-surface expression and enzymatic rates [18, 19]. Rather than a strictly linear cascade, these observations point to a reciprocal interplay between Na+/K+ ATPase and PKC signaling, in which disturbances in one pathway can trigger a feedback loop that affects the other. All these processes combined could also lead to alterations in oxidative stress.

In this context, tamoxifen—a selective estrogen receptor modulator with potent PKC inhibitory properties—has emerged as a promising antimanic agent with well-characterized translational relevance. Preclinically, tamoxifen has been shown to reverse manic-like behaviors in animal models, including amphetamine-induced hyperlocomotion [20] and ouabain-induced hyperactivity [21], while acting against oxidative stress parameters in rat brain [20, 22]. The antimanic efficacy of tamoxifen has been confirmed in multiple randomized, placebo-controlled trials, with a systematic review and meta-analysis of five RCTs demonstrating that tamoxifen significantly outperforms placebo in reducing manic symptom severity [23].

Oxidative stress is defined as an imbalance between the production of reactive oxygen species (ROS) and antioxidant capabilities [24]. A similar imbalance occurs with the production of nitrosants, such as nitric oxide, and antioxidants [25]. Both mechanisms are important hallmarks of BD pathophysiology [26] and exist in a bidirectional relationship with both Na+/K+ ATPase function [8, 27] and PKC signaling [20, 28], potentially acting as a systemic link that reinforces the dysregulation of both enzymes. Therefore, this study aimed to investigate the interactions between Na⁺/K⁺-ATPase inhibition and PKC signaling, as well as the associated oxidative stress, using a translational approach that combines preclinical (in vitro and in vivo) models and clinical data from patients with BD.

## Materials and Methods

### Chemicals and Reagents

All chemical reagents were purchased from commercial sources and used without further purification unless otherwise noted. Ouabain ≥ 95% - Sigma-Aldrich (O3125 - St. Louis, MO, USA); Tamoxifen ≥ 99% - Sigma-Aldrich (T5648 - St. Louis, MO, USA); Dulbecco’s Modified Eagle’s Medium - Thermo Fisher Scientific (Waltham, MA, USA); Ketamine and xylazine - Vetnil (Louveira, SP, Brazil); Tramadol hydrochloride - União Química Farmacêutica Nacional S. A. (São Paulo, Brazil); MgCl_2_, NaCl, KCl, and Tris from Neon (Suzano, SP, Brazil); PKC assay kit - Abcam (ab139437, Cambridge, UK); TBARS and 8-Isoprostane assay kits - Cayman (Ann Arbor, Michigan, USA); 4-Hydroxy-2-nonenal and 3-nitrotyrosine assay kits - Cell Biolabs (Cell Biolabs, Inc., San Diego, California, USA).

### In Vitro Study Protocol

Twenty 7-day-old mice (BALB/C strain) were obtained from the breeding colony at the University of Southern Santa Catarina (UNESC). Experimental procedures complied with the National Council for Animal Experimentation Control (CONCEA) guidelines. Furthermore, this study was approved by the local ethics committee (*Comissão de Ética no Uso de Animais da Universidade do Extremo Sul Catarinense - CEUA*) under the protocol number 51/2024. As the mice were 7 days postnatal, they were still with their mothers, being nursed, in a room at 21 ± 1 °C under a controlled 12-hour light/dark cycle (lights on at 7:00 am). The dams had free access to water and food.

Mice were killed by decapitation without anesthesia. The skull was opened, and the whole brain was dissected and cleaned. Immediately after the euthanasia of the animals, the brain was placed in a chamber with constant air flux for incubation. The brains from the mice were homogenized in phosphate-buffered saline (0.9%) and plated at a density of 10⁵ cells/cm² in 75 cm² culture flasks with Dulbecco’s Modified Eagle’s Medium supplemented with 10% fetal bovine serum and 20% antibiotics. Ouabain was immediately added, and 30 min later, tamoxifen, a PKC inhibitor (PKCi), was added to the medium, aiming to reach a 10^− 6^M concentration, based on the study by Nabha et al. [29]. The plate was kept in a carbon dioxide (CO_2_) incubator for 24 h. After that, samples were stored at − 80 °C for the subsequent analyses. The experimental groups were as follows: control (culture medium and sample); PKCi (culture medium, sample, and tamoxifen 10^− 6^M); a negative control or ouabain (culture medium, sample, and ouabain); and the group for the test of PKCi (culture medium, sample, ouabain, and PKCi); five animals were used per group. This initial in vitro study was designed to optimize animal usage, aligning with international ethical guidelines for reduction and refinement. Given that ICV administration is highly invasive, stressful, and associated with significant mortality, a preliminary in vitro screening was performed. Because these initial findings confirmed a significant interaction (see Results), the subsequent in vivo preclinical protocol was executed to further elucidate the behavioral and biochemical roles of Na+/K+ ATPase and PKC in BD. Rats were selected for the in vivo studies because the ICV ouabain injection model is widely standardized and validated in them for behavioral testing of mania and depression [27].

### In Vivo Preclinical Study Protocol

This protocol was conducted using adult male Wistar rats weighing 250–300 g obtained from the breeding colony at the University of Southern Santa Catarina (UNESC). Five animals were housed in a cage on a 12-hour light/dark cycle (lights on at 7:00 am), with free access to food and water. The rats were maintained at approximately 21 ± 1 °C with controlled humidity. All experimental procedures were carried out following CONCEA guidelines. Furthermore, this study was approved by the local ethics committee (CEUA).

Animals were subjected to anesthesia with ketamine (80 mg/kg body weight) and xylazine (10 mg/kg) intraperitoneally (i.p.) [20, 27]. In a stereotaxic apparatus, the skin covering the rat skull was removed, and a 27-gauge, 9 mm guide cannula was placed on the skull, according to the following coordinates: 0.9 mm posterior to bregma; 1.5 mm right from the midline; 1.0 mm above the lateral brain ventricle [30]. Through a 2-mm hole made in the cranial bone, a cannula was implanted 2.6 mm ventral to the superior surface of the skull and fixed with dental acrylic cement. Animals recovered from the procedure within four days. On each day of the recovery period, rats received tramadol hydrochloride (10 mg/kg body weight) subcutaneously every 12 h to mitigate postoperative pain, according to the manufacturer’s recommendation (União Química Farmacêutica Nacional S. A., São Paulo, Brazil). To validate the accuracy of the stereotaxic coordinates for the ICV injections, a pilot procedure was performed on the day of surgery using methylene blue dye (*n* = 2). Correct ventricular placement was confirmed by the diffusion of the dye through the ventricular system before proceeding with the experimental cohort.

**Fig. 1:**
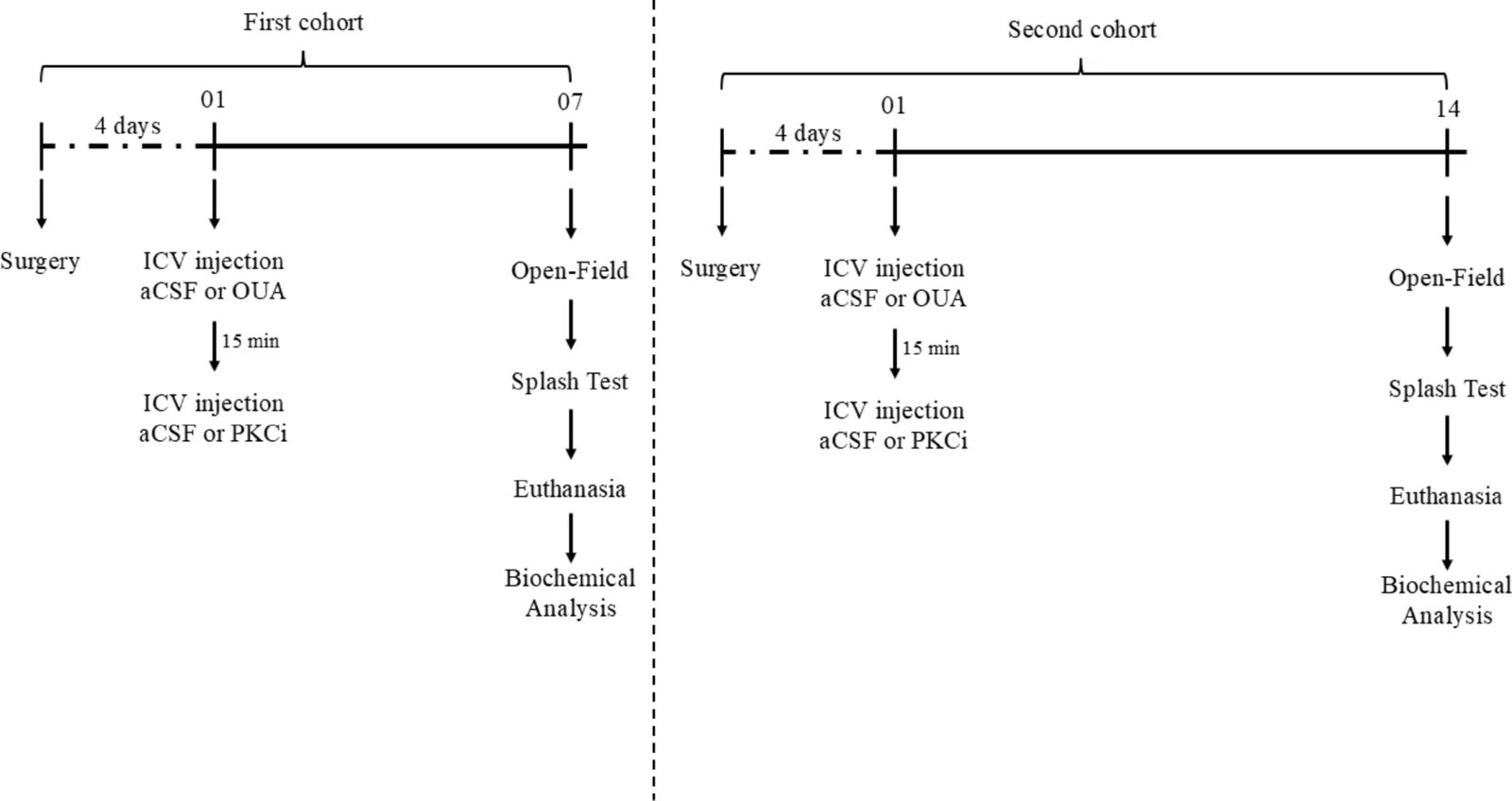
The scheme of the experimental timeline. ICV intracerebroventricular, PKCi protein kinase C inhibitor, OUA ouabain, aCSF artificial cerebrospinal fluid

In the present study, an in vivo model was used to simulate the management of an acute manic-like and depressive-like episode in animals. On the fourth day post-surgery, the animals were administered either a single intracerebroventricular (ICV) injection of 4 µL of ouabain (10^− 3^ M) dissolved in artificial cerebrospinal fluid (aCSF) or 4 µL of aCSF alone. A 30-gauge cannula was inserted into the guide cannula with a polyethylene tube connecting it to a microsyringe. The cannula infusion tip was positioned 1.0 mm beyond the cannula guide, precisely targeting the right lateral brain ventricle. At 15 min post-ouabain or aCSF infusion, tamoxifen (PKCi 10^− 6^ M) or aCSF (1 µL) was administered into the lateral ventricle. The experiment was divided into 2 different cohorts; the first cohort was evaluated 7 days after the ICV injection, and the second cohort was evaluated 14 days after the ICV injection. The animals were divided into four groups for the experimental procedure: group 1 received aCSF + aCSF; group 2 received aCSF + PKCi; group 3 received ouabain + aCSF; and group 4 received ouabain + PKCi (see Fig. 1).

### Behavioral Assessment

The different cohorts were subjected to the open-field and splash test, seven (first cohort) and fourteen days (second cohort) after the ICV injection. The researchers who analyzed the behavior tests were blinded to the experimental groups.0.

#### Open Field Test

The open-field test was used to assess the free movements of the animals and to evaluate the presence of hyperactivity, a key behavior associated with mania-like episodes. The task was performed in a 40 × 60 cm open field surrounded by 50-cm-high walls made of brown plywood with a frontal glass wall. The floor of the open field was divided into nine equal rectangles with black lines. A video camera was installed to record the animals’ behavior. Rats were placed individually in the open field for 5 min, and the following parameters were analyzed: number of crossings (times that the animal entered another square with all paws), number of rearings, and number of visits to the center of the apparatus. After each trial, the apparatus was cleaned with a 10% ethanol solution.

#### Splash Test

The rats were subjected to the splash test immediately after the open-field test, seven and fourteen days after ICV injection. This test consists of spraying a 10% sucrose solution on the dorsal coat of the rats [31]. The sucrose solution induces grooming behavior (nose/face grooming, head washing, and body grooming), which was observed for five minutes in the same apparatus as the open-field test. The time spent grooming is an index of self-care and motivational behavior, and a reduction of this behavior is associated with depressive-like episodes.

### Biochemical Analysis

Rats were submitted to euthanasia by decapitation, and the entire brain was removed, washed, and stored at − 80 °C. A previous report from our research group identified that Na+/K+ ATPase inhibition was more pronounced in the whole brain vs. specific brain regions [27]. With that in mind, in this study we decided to evaluate the biochemical alterations in the whole brain. Additionally, the biochemical analyses were performed on the two independent cohorts.

#### Na^+^/K^+^-ATPase Activity

The reaction mixture for the Na^+^/K^+^ ATPase activity assay contained 5.0 mM MgCl_2_, 80.0 mM NaCl, 20.0 mM KCl, and 40.0 mM Tris–HCl, pH 7.4, in a final volume of 200 µL. The reaction was initiated by adding ATP to a final concentration of 3.0 mM. Controls were carried out under the same conditions with the addition of 1.0 mM ouabain. Na+/K+-ATPase activity was calculated by the difference between the two assays according to the method of Wyse and coworkers [32]. Released inorganic phosphate (Pi) was measured using Chan and coworkers’ method [33]. The specific activity of the enzyme was expressed as nmol Pi released per min per mg of protein.

#### PKC Activity

The PKC activity was measured using commercially available kits (Abcam, ab139437; Cambridge, UK). Briefly, the homogenized samples were added to a pre-coated well in a microplate with a synthetic peptide identified as a substrate for PKC. After the phosphorylation of the enzyme by exogenous ATP, a polyclonal primary antibody recognizes the PKCp. Also, an HRP-conjugated antibody was added to the well. Finally, a TMB substrate was added, and the plate was incubated for 30–60 min. Lastly, the plate was read at 450 nm and the intensity of the signal is proportional to the activity of PKC. Purified active PKC was used as a positive control. The activity of the enzyme was expressed in pmol/min/mg protein.

#### Evaluation of Oxidative and Nitrosative Damage Parameters in the Cerebral structures

The thiobarbituric acid reactive species (TBARS) Assay Kit (Cayman; catalog no. 10009055) was used for the direct quantitative measurement of the malondialdehyde (MDA) level in the samples of brain tissue. 4-Hydroxy-2-nonenal (4-HNE) content was quantified using the kit provided by Cell Biolabs (Cell Biolabs, Inc., San Diego, California, USA; catalog no. STA-338). Protein adducts arising from the modification of cysteine, histidine, or lysine residues by 4-HNE were quantified using Kimura and coworkers’ [34] methods. 8-Isoprostane content was measured using the ACE™ Competitive EIAs Kit (Cayman; catalog no. 516351) with 8-isoprostane-acetylcholinesterase (EC 3.1.1.7) as a tracer and 8-isoprostane-specific antiserum. Protein oxidation parameters Carbonyl group content was measured using a specific kit provided by Cell Biolabs (OxiSelect™ Protein Carbonyl ELISA Kit; catalog no. STA-310). For the quantitative measurement of 3-nitrotyrosine (3-nitro) (Enzyme-Linked Immunosorbent Assay) in the brain, ELISA kit Cell Biolabs, INC. (STA-305-5) was used.

### Clinical Study Protocol

Clinical data were derived from a secondary analysis of a previously published sample [8]. Participants were recruited from the Psychiatry Clinic at UNESC in Criciúma, Santa Catarina, Brazil. The study encompassed 36 individuals diagnosed with BD type I, with 7 experiencing the euthymic phase, 23 in a state of depression, 3 in a manic phase, and 3 in a mixed manic condition. Additionally, 46 healthy control (HC) participants matched in age and sex underwent assessment. Per the Diagnostic and Statistical Manual of Mental Disorders-IV (DSM-IV), these controls displayed no ongoing or past Axis I disorders. Psychiatric diagnoses were based on clinical interviews and were confirmed with the Structured Clinical Interview for DSM-IV Axis I Disorders (SCID-I) [35]. Table 1 shows further details regarding the sociodemographic data from the study population.

**Table 1:** Sample demographics

	Mean ± SD; *n* (%)		Statistics
	BD	HC	
	*n* = 36	*n* = 46	
Age (years)	41.55 ± 12.76	37.65 ± 13.47	U = 678,00, *p* = 0.161
Sex			
Male	7 (19.4)	10 (21.7)	x^2^ = (1) 0.79, *p* = 0.065
Female	29 (80.6)	36 (78.3)	
BMI (kg/m^2^)	27.34 ± 7.38	26.11 ± 7.25	U = 756.00, *p* = 0.064
Years of study	12.75 ± 5.07	15.58 ± 7.11	U = 1.022,50, *p* = 0.069
Current smoking	11 (30.6)	8 (17.4)	x^2^ = (1) 1.96, *p* = 0.161
Family psychiatric history	24 (66.7)	24 (52.2)	x^2^ = (1) 1.78, *p* = 0.186
BD subtype			
Type I	30 (83.3)	–	–
Type II	5 (13.9)	–	–
Cyclothymic	1 (2.8)	–	–
Current medications			
Lithium	11 (30.6)	–	–
Anticonvulsant	23 (63.9)	–	–
Antidepressants	21 (61.2)	–	–
Antipsychotic	20 (55.5)	–	–
Anxiolytic	8 (22.2)	–	–
Psychoanaleptics	2 (5.6)	–	–
Anticholinergic	1 (2.8)	–	–

Sample demographics from the present study. Data from Valvassori et al. [8]. *BD* bipolar disorders, *HC* healthy control, *SD* standard deviation, *BMI* body mass index. Numerical variables [Mean ± SD] were analyzed using Mann-Whitney U test and categorical variables [n (%)] were analyzed using chi square

The criteria for exclusion across all samples included organic psychosis, neurological conditions, unstable medical issues impacting brain function, and age falling outside the range of 18 to 65. After explaining the purpose of the study, informed consent was obtained from each participant. Additionally, 20 mL of blood was collected from each BD patient and HC via venipuncture into heparin tubes, EDTA tubes, and tubes without anticoagulants. The peripheral blood was processed for evaluation of Na+/K+-ATPase activity (erythrocyte membrane), PKC activity (erythrocytes), and oxidative stress parameters (serum), using the same protocols described previously. Further details of the methodology can be found in Valvassori et al. [8].

The Regional Ethics Committee, *Comitê de Ética em Pesquisa e Humanos*, approved the current study with the assigned protocol number 2,928,502.

### Statistical Analysis

#### Preclinical Data

Shapiro-Wilk’s test was used to evaluate the normality of the data, and all preclinical data presented a normal distribution. Two-way ANOVA followed by Tukey’s post hoc test was used to evaluate the differences between the groups. Pearson’s correlation was used to evaluate the correlation between PKC vs. Na + K+ATPase data is presented as mean ± standard deviation (SD). A p-value ≤ 0.05 was considered statistically significant. All analyses were performed in GraphPad Prism^®^ 10.

#### Clinical Data

Descriptive statistics and correlations were computed for clinical data to examine the association and differences between the BD and healthy control groups. The normality of data distribution was assessed using the Shapiro-Wilk test. Student’s t-test or Mann-Whitney U test was used to identify differences in continuous variables (Na+/K+-ATPase, PKC, 4-HNE, 8-ISO, Carbonyl, 3-Nitrotyrosine, and TBARS) between the BD and healthy control groups. Spearman correlations were computed to assess the association between Na+/K+-ATPase, PKC, and oxidative stress markers in BD patients. A linear regression analysis was performed to investigate the effects of Na+/K+-ATPase on PKC activity and oxidative stress levels in BD patients. There were no missing data across the variables of interest in the study. *P*-values < 0.05 were taken as statistically significant. All analyses and data organization were conducted in SPSS 23.0.

## Results

### Preclinical In Vitro Results

**Fig. 2:**
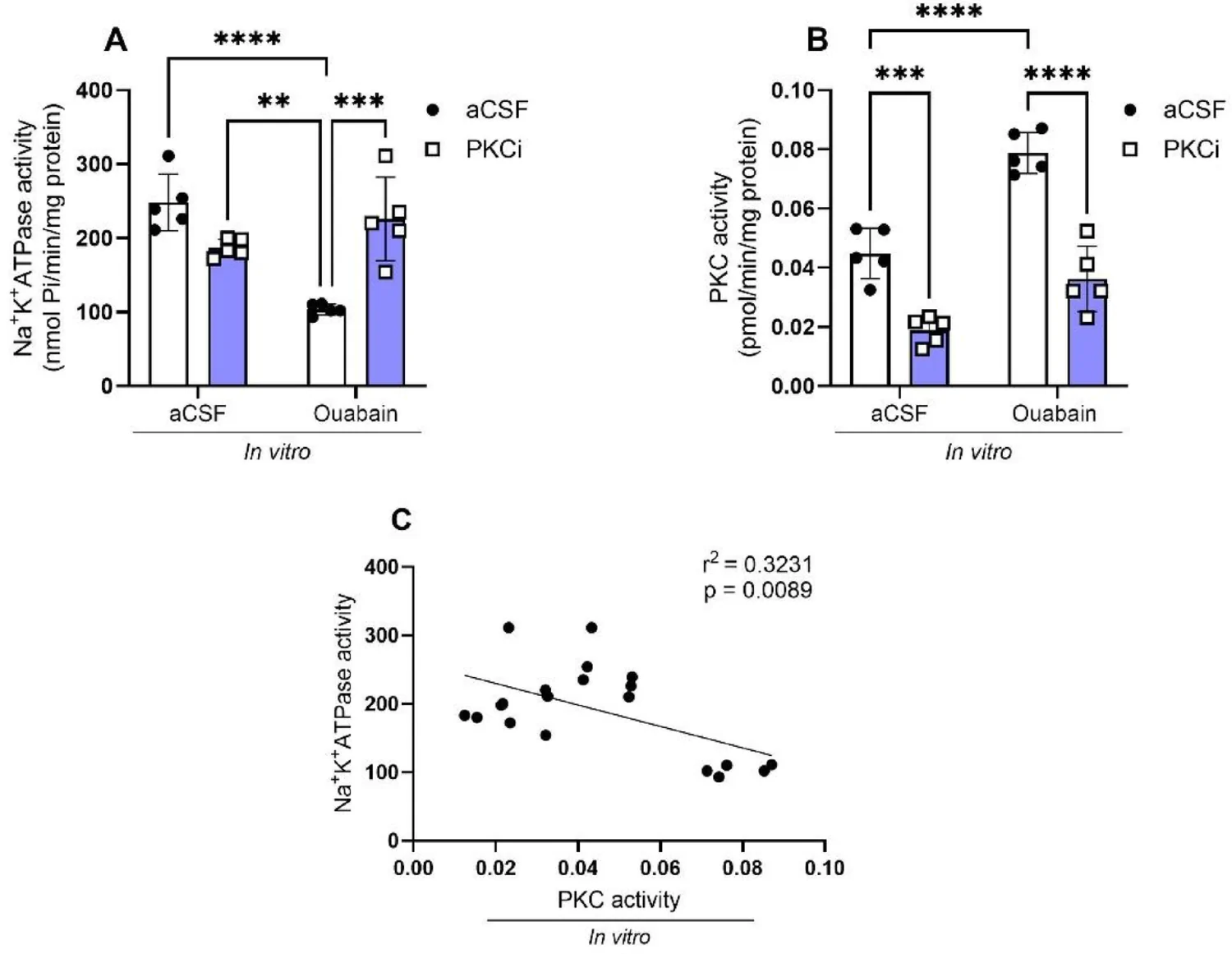
Effects of the Protein Kinase C inhibitor (PKCi) on the activity of Na^+^K^+^-ATPase and PKC, and the correlation between the two, in the brain of Balb/C mice exposed to OUA in vitro. Data is presented as mean ± standard deviation. Two-way ANOVA followed by Tukey’s post hoc test was used to evaluate the difference between the groups and Pearson Correlation for the correlation. * *p* ≤ 0.05; ** *p* ≤ 0.01; *** *p* ≤ 0.001; **** *p* ≤ 0.0001

Figure 2 demonstrates the PKCi effects on the Na^+^/K^+^-ATPase and PKC activity in brain cells of Balb/C mice exposed to OUA in vitro. Figure 2A demonstrates the activity of Na^+^/K^+^-ATPase after the treatment with PKCi in brain cells of Balb/C exposed to OUA in vitro. Two-way ANOVA indicated a significant variance between Control vs. OUA [F (1, 16) = 11.34, *p* = 0.003], and the interaction between the two [F (1, 16) = 34.70, *p* < 0.001], but not Control vs. PKCi [F (1, 16) = 3.789, *p* = 0.069]. According to Tukey’s post hoc, OUA reduced the activity of Na^+^/K^+^-ATPase in the brain cell in vitro compared to the control [*p* < 0.001]. Also, PCKi reverted the effects of OUA on the Na^+^/K^+^-ATPase activity [*p* < 0.001] without reducing this enzyme activity in the Control + PKCi group [*p* = 0.057].

Two-way ANOVA revealed a significant alteration in the activity of PKC (Fig. 2B) between Control vs. OUA [F (1, 16) = 49.45, *p* < 0.001], Control vs. PKCi [F (1, 16) = 88.30, *p* < 0.001], and the interaction [F (1, 16) = 5.210, *p* = 0.036]. Tukey’s post hoc indicated that PCKi by itself reduces the activity of PKC when compared to the control [*p* < 0.001]. In addition, OUA significantly increased the activity of PKC when compared to control [*p* < 0.001]. Interestingly, PCKi reduced the activity of PCK in the OUA group, but not beyond the control group [vs. OUA *p* < 0.001; vs. Control *p* = 0.369]. In addition, a Pearson correlation indicated a very weak negative correlation between PCK activity and Na^+^/K^+^-ATPase activity in brain cells of Balb/C in vitro [r² = 0.32; *p* = 0.009], represented in Fig. 2C.

**Fig. 3:**
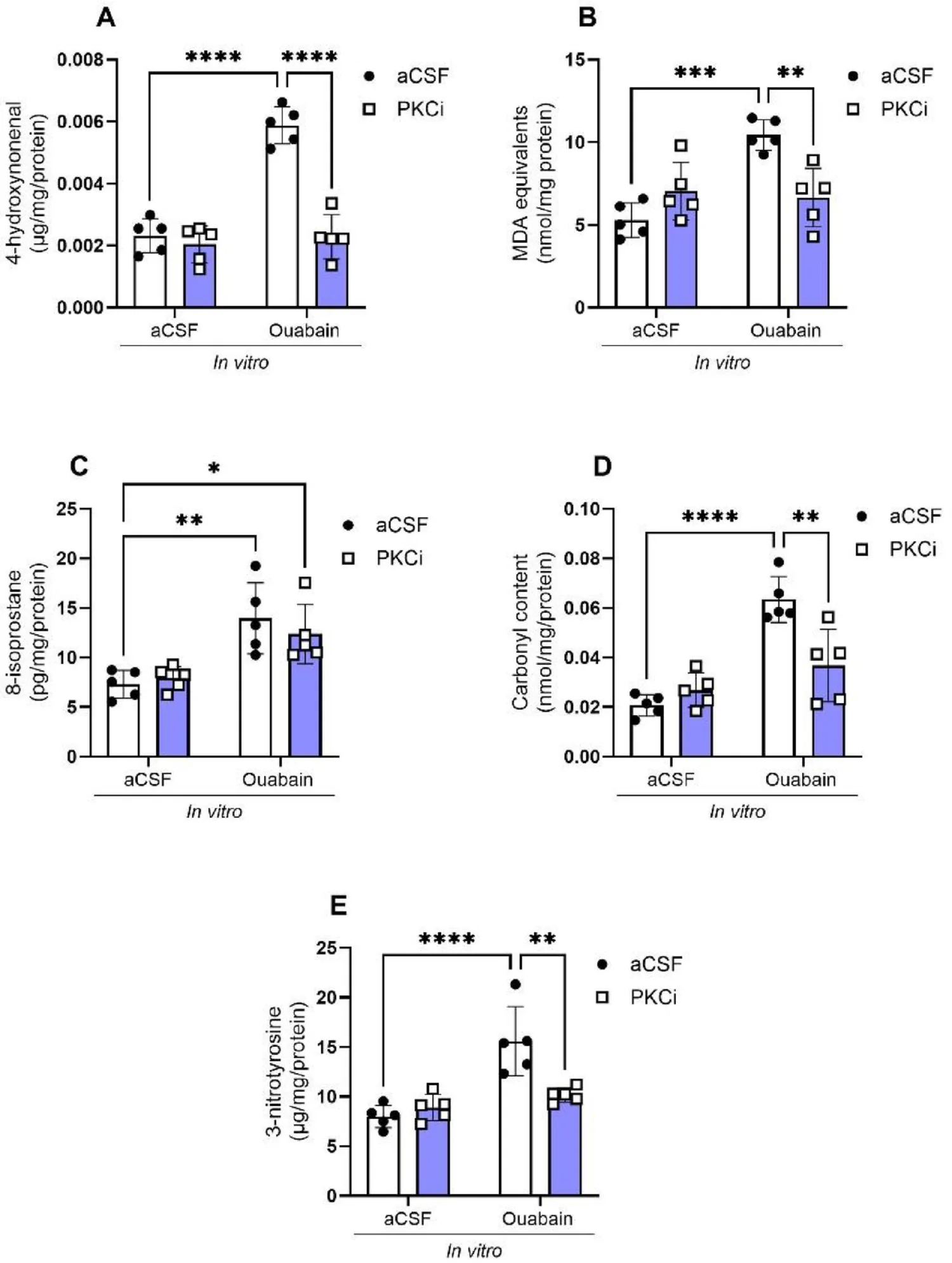
Effects of the Protein Kinase C inhibitor (PKCi) on the levels of 4-HNE, MDA, 8-ISO, Carbonyl, and 3-nitro in the brain of Balb/C mice exposed to OUA in vitro. Data is presented as mean ± standard deviation. Two-way ANOVA followed by Tukey’s post hoc test was used to evaluate the difference between the groups. * *p* ≤ 0.05; ** *p* ≤ 0.01; ***p ≤ 0.001; ****p ≤ 0.0001

The effects of the PKCi on oxidative stress parameters in brain cells exposed to OUA in vitro are presented in Fig. 3. The levels of 4-HNE (Fig. 3A), according to Two-way ANOVA, showed a significant alteration in Control vs. OUA [F (1, 16) = 47.71, *p* < 0.001], Control vs. PKCi [F (1, 16) = 49.39, *p* < 0.001], and the interaction between the two [F (1, 16) = 36.10, *p* < 0.001]. Tukey’s post hoc revealed that PKCi alone did not significantly interfere with the levels of 4-HNE [*p* = 0.887]. On the other hand, OUA significantly increased the levels of this oxidative damage marker [*p* < 0.001], which was reverted by the PKCi [*p* < 0.001]. Two-way ANOVA also revealed that the levels of MDA (Fig. 3B) were significantly different between Control vs. OUA [F (1, 16) = 14.22, *p* = 0.001], and the interaction [F (1, 16) = 19.22, *p* < 0.001], but not Control vs. PKCi [F (1, 16) = 2.607, *p* = 0.125]. According to Tukey’s post hoc test, OUA increased the levels of MDA [*p* < 0.001], which was reduced by PKCi [*p* = 0.003]. On the contrary, the levels of 8-ISO (Fig. 3C) were only significantly different between Control vs. OUA [F (1, 16) = 24.44, *p* < 0.001], and not with Control vs. PKCi [F (1, 16) = 0.19, *p* = 0.664] and the interaction [F (1, 16) = 0.94, *p* = 0.345] according to Two-way ANOVA. Tukey’s post-hoc revealed a significant increase in the levels of 8-ISO caused by OUA [*p* = 0.003], which was not reverted by PKCi [*p* = 0.751].

Furthermore, the levels of carbonyl and 3-nitro were also evaluated. Two-way ANOVA revealed that the levels of carbonyl (Fig. 3D) presented significant variance between Control vs. OUA [F (1, 16) = 38.08, *p* < 0.0001], Control vs. PKCi [F (1, 16) = 5.81, *p* = 0.028], and the interaction [F (1, 16) = 14.60, *p* = 0.001]. Tukek’s post hoc demonstrated that OUA significantly increased the levels of carbonyl [*p* < 0.001], reverted by PKCi [*p* = 0.002]. It is important to note that PKCi alone did not interfere with this oxidative stress marker [*p* = 0.753]. Regarding the levels of 3-nitro (Fig. 3E), Two-way ANOVA showed significant alteration in Control vs. OUA [F (1, 16) = 24.76, *p* < 0.001], Control vs. PKCi [F (1, 16) = 6.498, *p* = 0.021] and the interaction [F (1, 16) = 12.76, *p* = 0.002]. Tukey’s post hoc indicated a significant increase in the levels of 3-nitro caused by OUA [*p* < 0.001], reverted by PKCi [*p* = 0.002]. In addition, the PKCi caused no alteration by itself [*p* = 0.886].

### Preclinical In Vivo Results

**Fig. 4:**
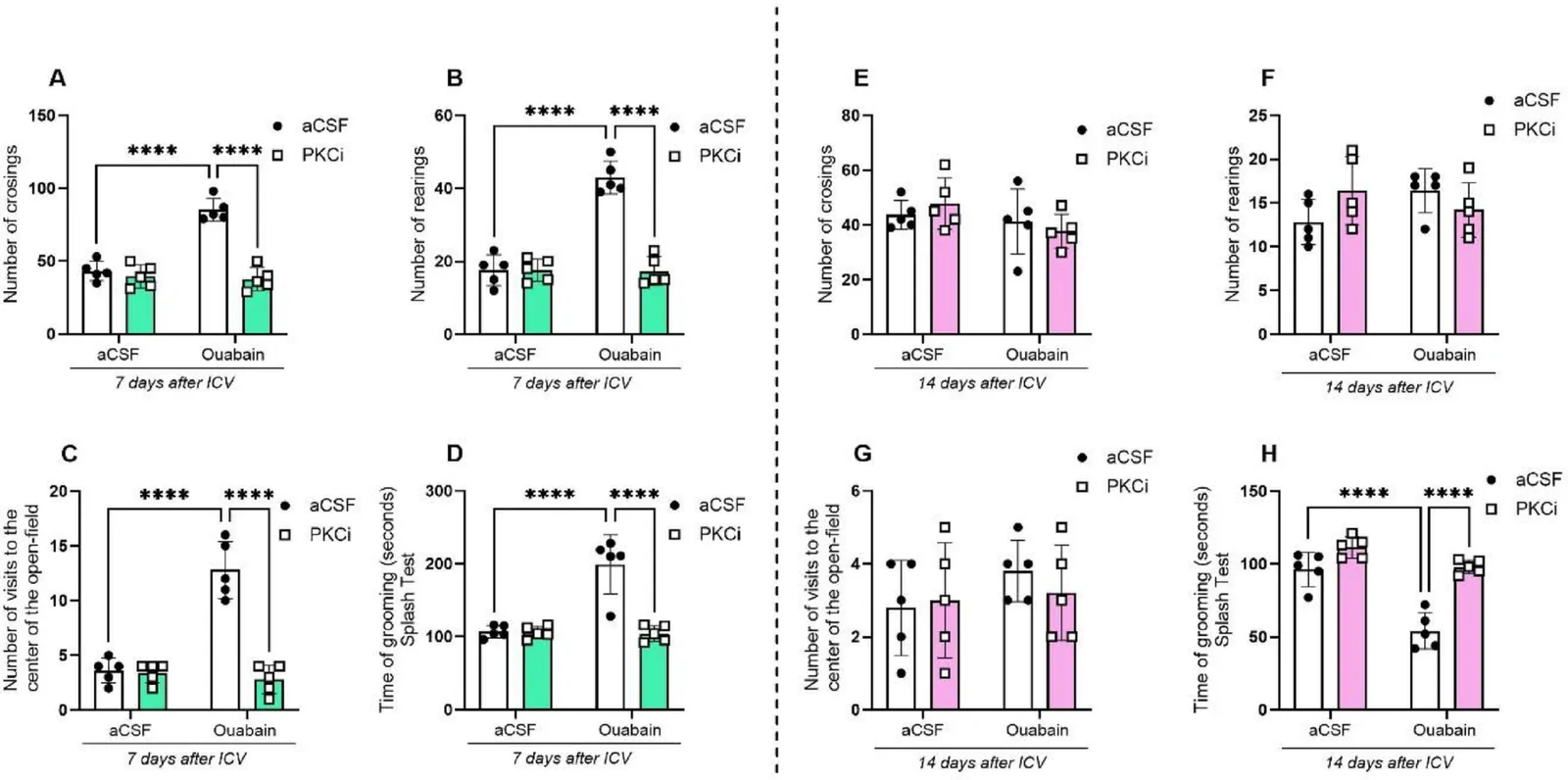
Effects of the Protein Kinase C inhibitor (PKCi) on the number of crossing, rearing, and visits to the center (open field), and time of grooming (splash test) 7 days (A-D), and 14 days (E-H) after the ICV of OUA in male Wistar rats. Data is presented as mean ± standard deviation. Two-way ANOVA followed by Tukey’s post hoc test was used to evaluate the difference between the groups. * *p* ≤ 0.05; ** *p* ≤ 0.01; ***p ≤ 0.001; ****p ≤ 0.0001

The results from the open field test for 7 days after ICV, number of crossings, rearing, and visits to the center of the apparatus, are represented in Fig. 4A–C. On 7 days after the ICV, in all these parameters, Two-way ANOVA revealed a significant difference between aCSF vs. OUA [Crossing: F (1, 16) = 35.15, *p* < 0.001; Rearing: F (1, 16) = 50.48, *p* < 0.001; Center: F (1, 16) = 35.22, *p* < 0.001], aCSF vs. PKCi [Crossing: F (1, 16) = 35.15, *p* < 0.001; Rearing: F (1, 16) = 52.10, *p* < 0.001; Center: F (1, 16) = 49.54, *p* < 0.001], and the interaction between the two [Crossing: F (1, 16) = 41.72, *p* < 0.001; Rearing: F (1, 16) = 52.10, *p* < 0.001; Center: F (1, 16) = 45.73, *p* < 0.001]. According to Tukey’s post hoc, OUA increased the number of crossings [*p* < 0.001], rearing [*p* < 0.001], and visits to the center [*p* < 0.001]. In addition, PKCi reverted all the effects of OUA in the parameters [crossing *p* < 0.0001; rearing *p* < 0.001; center *p* < 0.001], but did not interfere alone [crossing *p* = 0.875; rearing *p* > 0.999; center *p* = 0.997]. Moreover, a splash was also performed after the open field test, and the time of grooming (Fig. 4D) was analyzed. Two-way ANOVA revealed that aCSF vs. OUA [F (1, 16) = 21.71, *p* < 0.001], aCSF vs. PKCi [F (1, 16) = 24.06, *p* < 0.001], and the interaction [F (1, 16) = 23.26, *p* < 0.001], were statistically different. Tukey’s post hoc pointed out that OUA significantly increased the time of grooming [*p* < 0.001], reverted by PKCi [*p* < 0.001].

Furthermore, a behavior analysis 14 after the ICV was also performed. Data from Two-way ANOVA revealed no significant differences in the number of crossing [aCSF vs. OUA: F (1, 16) = 2.67, *p* = 0.121; aCSF vs. PKCi: F (1, 16) = 2.67, *p* = 0.121; interaction: F (1, 16) = 1.02, *p* = 0.326], rearing [aCSF vs. OUA: F (1, 16) = 0.258, *p* = 0.618 aCSF vs. PKCi: F (1, 16) = 0.258, *p* = 0.618; interaction: F (1, 16) = 4.42, *p* = 0.051], and visits to the center [aCSF vs. OUA: F (1, 16) = 1.091, *p* = 0.3118; aCSF vs. PKCi: F (1, 16) = 0.1212, *p* = 0.732; interaction: F (1, 16) = 0.484, *p* = 0.496], in the open field test (Fig. 4E-G). Lastly, the splash was evaluated as well. According to Two-way ANOVA, the time of grooming (Fig. 4H) showed significant differences in aCSF vs. OUA [F (1, 16) = 41.91, *p* < 0.0001], aCSF vs. PKCi [F (1, 16) = 46.87, *p* < 0.001], and the interaction [F (1, 16) = 11.25, *p* = 0.004]. Tukey’s post hoc demonstrated that PKCi alone did not interfere with the time of grooming [*p* = 0.103]. In addition, OUA reduced grooming [*p* < 0.001], which was reverted by PKCi [*p* < 0.001].

**Fig. 5:**
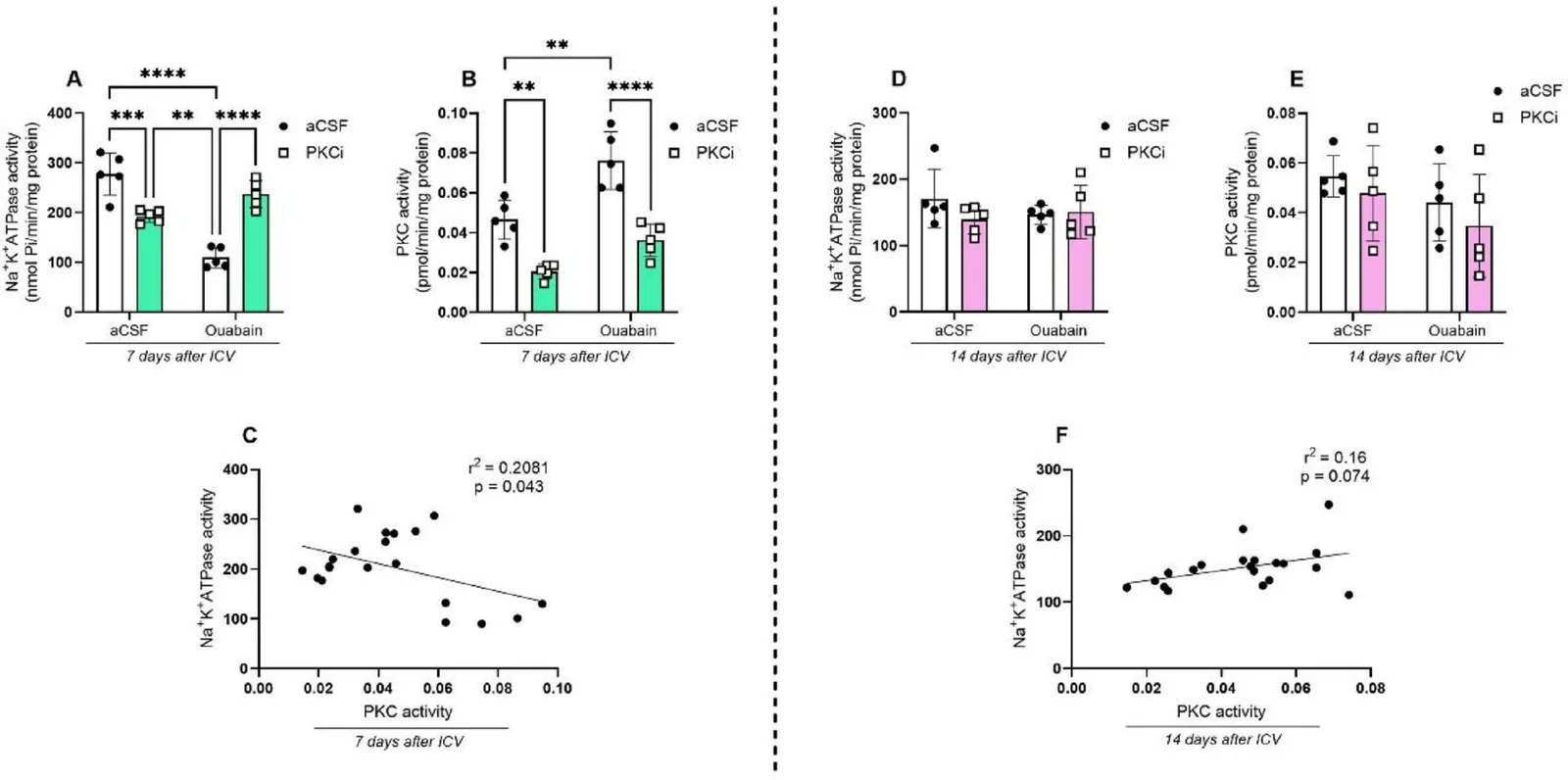
Effects of the Protein Kinase C inhibitor (PKCi) on the activity of Na^+^K^+^-ATPase and PKC, and the correlation between the two, in Wistar rats after 7 (**A-C**) and 14 days (D-F) of ICV of OUA. Data is presented as mean ± standard deviation. Two-way ANOVA followed by Tukey’s post hoc test was used to evaluate the difference between the groups and Pearson Correlation for the correlation. * *p* ≤ 0.05; ** *p* ≤ 0.01; ***p ≤ 0.001

Similar to what was performed in the in vitro protocol, the activity of Na+/K+-ATPase and PKC were measured in vivo to evaluate further the effects of PKCi in the brain exposed to OUA. 7 days after the ICV of OUA, the activity of Na+/K+-ATPase (Fig. 5A) was measured in the whole brain and Two-way ANOVA revealed significant differences between aCSF vs. OUA [F (1, 16) = 24.84, *p* < 0.001], and the interaction [F (1, 16) = 72.78, *p* < 0.001], but not aCSF vs. PKCi [F (1, 16) = 2.97, *p* = 0.103]. Tukey’s post hoc demonstrated a significant reduction in the activity of Na+/K+-ATPase caused by the PKCi [*p* = 0.011] and OUA [*p* < 0.001], both alone. Interestingly, the reduction caused by OUA was significantly stronger than the inhibition caused by PKCi [*p* < 0.001]. Also, when combined, the PKCi reverted the Na+/K+-ATPase inhibition caused by OUA [vs. OUA *p* < 0.001; vs. aCSF *p* = 0.192]. Regarding the PKC activity (Fig. 5B), Two-way ANOVA revealed a significant difference in aCSF vs. OUA [F (1, 16) = 26.85, *p* < 0.001], and aCSF vs. PKCi [F (1, 16) = 56.90 *p* < 0.001], but not the interaction [F (1, 16) = 2.55, *p* = 0.129]. Tukey’s post hoc showed that the PKC inhibitor reduced the activity of PKC [*p* = 0.003]. Also, OUA increased the activity of this enzyme [*p* = 0.001], and PKCi reverted these effects without differing from the aCSF [vs. OUA *p* < 0.001; vs. aCSF *p* = 0.370]. Pearson correlation (Fig. 5C) also indicated a very weak negative correlation between PKC and Na+/K+-ATPase activity [*r* = 0.208; *p* = 0.043].

The same parameters were also evaluated 14 days after the ICV of OUA. Interestingly, Na+/K+-ATPase (Fig. 5D) and PKC activity (Fig. 5E) showed no significant difference caused by either OUA or PKCi according to Two-way ANOVA [Na+/K+-ATPase - aCSF vs. OUA: F (1, 16) = 0.191, *p* = 0.668; aCSF vs. PKCi: F (1, 16) = 0.930, *p* = 0.349; Interaction: F (1, 16) = 1.612, *p* = 0.222; PKC - aCSF vs. OUA: F (1, 16) = 2.471, *p* = 0.135; aCSF vs. PKCi: F (1, 16) = 1.180, *p* = 0.293; Interaction: F (1, 16) = 0.028, *p* = 0.867]. Pearson correlation analysis between PCK and Na+/K+-ATPase (Fig. 5F) also showed no significant correlation [*r* = 0.16; *p* = 0.074].

**Fig. 6:**
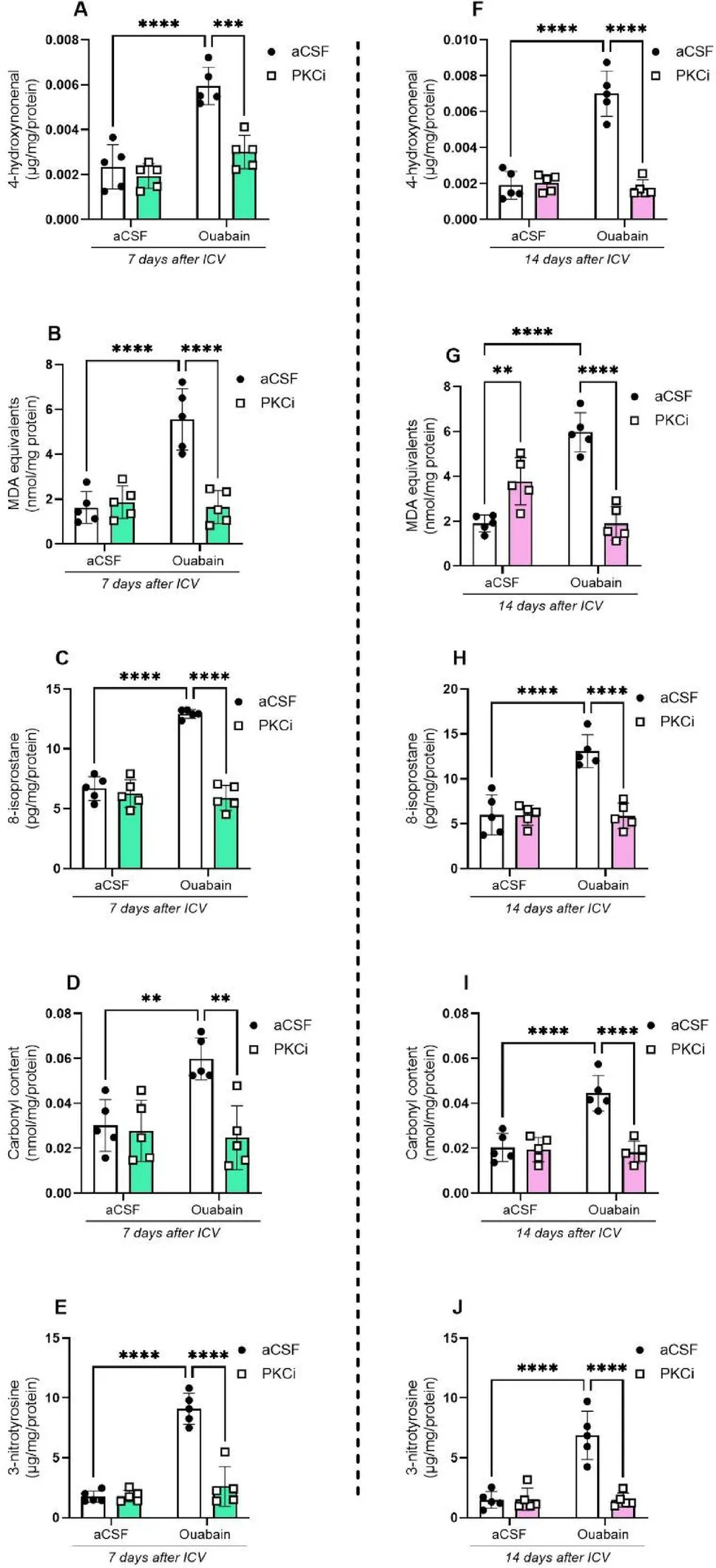
Effects of the Protein Kinase C inhibitor (PKCi) on the levels of 4-HNE, MDA, 8-ISO, Carbonyl, and 3-nitro in Wistar rats after 7 (**A-E**) and 14 days (**F-J**) of ICV of OUA. Data is presented as mean ± standard deviation. Two-way ANOVA followed by Tukey’s post hoc test was used to evaluate the difference between the groups. * *p* ≤ 0.05; ** *p* ≤ 0.01; ***p ≤ 0.001;****p ≤ 0.0001

The oxidative and nitrosative stress markers were also evaluated in the in vivo preclinical protocol 7 and 14 days after the ICV of OUA and PKCi. 7 days after the ICV, Two-way ANOVA revealed that, for the levels of 4-HNE (Fig. 6A), MDA (Fig. 6B), 8-ISO (Fig. 6C), Carbonyl (Fig. 6D), and 3-nitro (Fig. 6E) the variance between aCSF vs. OUA [4-HNE F (1, 16) = 43.10, *p* < 0.001; MDA F (1, 16) = 19.91, *p* < 0.001; 8-ISO F (1, 16) = 47.89, *p* < 0.001; Carbonyl F (1, 16) = 5.83, *p* = 0.028; 3-nitro F (1, 16) = 67.14, *p* < 0.001], aCSF vs. PKCi [4-HNE F (1, 16) = 22.19, *p* < 0.001; MDA F (1, 16) = 19.60, *p* < 0.001; 8-ISO F (1, 16) = 77.66, *p* < 0.001; Carbonyl F (1, 16) = 11.53, *p* = 0.003; 3-nitro F (1, 16) = 41.91, *p* < 0.001], and the interaction [4-HNE F (1, 16) = 12.76, *p* = 0.002; MDA F (1, 16) = 25.07, *p* = 0.001; 8-ISO F (1, 16) = 60.93, *p* < 0.001; Carbonyl F (1, 16) = 8.78, *p* = 0.009; 3-nitro F (1, 16) = 43.43, *p* < 0.001] were statistically different. Tukey’s post hoc demonstrated that OUA increased the levels of 4-HNE [*p* < 0.001], MDA [*p* < 0.001], 8-ISO [*p* < 0.001], carbonyl [*p* = 0.007], and 3-nitro [*p* < 0.001]. In addition, PKCi reverted all these effects on oxidative and nitrosative markers [4-HNE *p* = 0.001; MDA *p* < 0.001; 8-ISO *p* < 0.001; Carbonyl *p* = 0.001; 3-nitro *p* < 0.001].

Similar to what happened 7 days after the ICV, according to Two-way ANOVA, the levels of 4-HNE [aCSF vs. OUA F (1, 16) = 44.50, *p* < 0.001; aCSF vs. PKCi F (1, 16) = 50.47, *p* < 0.001; interaction F (1, 16) = 55.56, *p* < 0.001], MDA [aCSF vs. OUA F (1, 16) = 9.265, *p* = 0.007; aCSF vs. PKCi F (1, 16) = 9.265, *p* = 0.007; interaction F (1, 16) = 68.39, *p* < 0.001], 8-ISO [aCSF vs. OUA F (1, 16) = 21.47, *p* = 0.003; aCSF vs. PKCi F (1, 16) = 22.89, *p* < 0.001; interaction F (1, 16) = 22.11, *p* < 0.001], carbonyl [aCSF vs. OUA F (1, 16) = 16.99, *p* < 0.001; aCSF vs. PKCi F (1, 16) = 23.75, *p* < 0.001; interaction F (1, 16) = 20.70, *p* < 0.001], and 3-nitro [aCSF vs. OUA F (1, 16) = 24.60, *p <* 0.001; aCSF vs. PKCi F (1, 16) = 24.58, *p* < 0.001; interaction F (1, 16) = 25.70, *p* < 0.001] presented significantly different variance between groups 14 days later (Fig. 6F-J). In addition, Tukey’s post hoc showed a significantly increased level of these parameters caused by OUA [4-HNE *p* < 0.001, MDA *p* < 0.001, 8-ISO *p* < 0.001, Carbonyl *p* < 0.001, 3-nitro *p* < 0.001], which were reverted by PKCi [4-HNE *p* < 0.001, MDA *p* < 0.001, 8-ISO *p* < 0.001, Carbonyl *p* < 0.001, 3-nitro *p* < 0.001]. Interestingly, PKCi alone increased the levels of MDA 14 days after the ICV [*p* = 0.009].

### Clinical Results

The study sample comprised 82 participants (36 BD patients and 46 healthy controls). Individuals with BD had higher PKC activity (mean = 3.00, SD = 3.30 pmol/min/mg/protein) than healthy controls (mean = 1.90, SD = 0.64 pmol/min/mg/protein; U = 566.50; *p* = 0.015) in the erythrocytes. Additionally, in a previous report from our research group [8], we found that individuals with BD had higher serum levels of 4-HNE, Carbonyl, 3-nitro, and TBARS. Also, BD had lower erythrocyte Na+/K+-ATPase activity than healthy controls.

Bivariate correlations between all study variables in the BD group are presented in Table 2. PKC was positively correlated with 4-HNE (*r* = 0.718; *p* < 0.001), 8-ISO (*r* = 0.689; *p* < 0.001), and Carbonyl (*r* = 0.249; *p* < 0.05).

**Table 2:** Bivariate correlations between all study variables (*n* = 36)

	PKC activity	
	*r*	*p*-value
Na + K+ATPase	− 0.109	0.525
4-HNE (mg/ml)	0.868	**< 0.001**
8-ISO (pg/ml)	0.797	**< 0.001**
Carbonyl (nM)	0.354	**0.034**
3-Nitrotyrosine (µg/mL)	− 0.015	0.932
TBARS (nM)	− 0.094	0.587

*BD* bipolar disorders, *HC* healthy controls, *PKC* protein kinase C, *4-HNE* 4-hydroxy-2-nonenal, *8-ISO* 8-isoprostane, *TBARS* thiobarbituric acid reactive substances. Bold number indicates significance (*p* < 0.05)

Table 3 presented detailed results of the analyses of regression linear examining Na+/K+-ATPase activity such outcome in BD patients. Na+/K+-ATPase activity showed a statistically significant inverse relationship with 4-HNE (F (1, 34) = 4.64, *p* = 0.038; *R*^*2*^ adjusted = 0.09) and with carbonyl (F (1, 34) = 11.436, *p* = 0.002; adjusted *R*^*2*^ = 0.23). There was no statistical relationship with the other dependent variables.

**Table 3:** Regression outcomes for Na+/K+-ATPase as predictor (*n* = 36)

		Standardized β	t	*p*	*R*^2^ adjusted
PKC activity	(Constant)		0.184	0.855	
	Na^+^K^+^ATPase	0.050	0.292	0.772	− 0.03
4-HNE (mg/ml)	(Constant)		4.97	< 0.001	
	Na^+^K^+^ATPase	− 0.347	−2.156	**0.038**	0.09
8-ISO (pg/ml)	(Constant)		2.998	0.005	
	Na^+^K^+^ATPase	− 0.138	− 0.812	0.423	− 0.01
Carbonyl (nM)	(Constant)		9.909	< 0.001	
	Na^+^K^+^ATPase	− 0.502	−3.382	**0.002**	0.23
3-Nitrotyrosine (µg/mL)	(Constant)		6.084	< 0.001	
	Na^+^K^+^ATPase	− 0.030	− 0.177	0.860	− 0.028
TBARS (nM)	(Constant)		4.36	< 0.001	
	Na^+^K^+^ATPase	0.010	0.061	0.952	− 0.029

The constant corresponds to the model intercept, i.e., the expected value of the outcome when Na⁺K⁺ATPase activity equals zero. Its *p*-value does not indicate an association between variables. Bold number indicates significance (*p* < 0.05)

*PKC* protein kinase C, *4-HNE* 4-hydroxy-2-nonenal, *8-ISO* 8-isoprostane, *TBARS* thiobarbituric acid reactive substances

## Discussion

BD is a complex and multifactorial disorder, with pathophysiological factors such as Na + K+ATPase and PKC characterizing the interaction between these two enzymes in the context of BD. Preclinical studies were performed in vitro, and an in vivo study was performed. OUA, a Na + K+ATPase inhibitor, did in fact reduce the activity of this enzyme in both models. It is important to reiterate that OUA, in our in vitro protocol, demonstrated the same biochemical effects observed in a previous study using animal models of mania and BD [27]. In addition, PKCi reduced PKC activity, an expected result given the inhibitory effects of tamoxifen [36]. As demonstrated herein, OUA induced both mania-like and depressive-like behaviors in the in vivo protocol. Specifically, 7 days after the ICV injection, OUA induced a mania-like phenotype, characterized by increased crossings (locomotion), rearings (exploratory behavior), and visits to the center of the apparatus (risk-taking behavior) in the open field test. Additionally, OUA-treated animals showed increased grooming time in the splash test at this same time point. Rather than an anti-anhedonic effect, this behavior is most likely linked to the model’s characteristic hyperactivity. However, 14 days post-ICV, the animals displayed no locomotor alterations in the open field, but exhibited a significant reduction in grooming time during the splash test. This shift is consistent with a depressive-like phenotype and aligns with previous findings from our research group. A more detailed discussion and revision of this model can be found in Valvassori et al., [27], and Possamai-Della et al., [37].

When administered alone, OUA increased PKC activity in both in vitro and in vivo preclinical protocols 7 days post-ICV. Previous studies have demonstrated interactions between Na+/K+-ATPase and various intracellular pathways, including Ras/Raf/MEK/ERK1/2, mitochondrial ROS production, and Src-dependent PKC activation [14, 38, 39]. Specifically, the binding of OUA to Na+/K+-ATPase activates and recruits Src, initiating a signaling cascade in which PKC serves as a downstream target [38]. Through the Src-dependent activation of the epidermal growth factor receptor (EGFR), OUA-bound Na+/K+-ATPase stimulates the PLC/PIP_2_/DAG pathway and elevates intracellular Ca²^+^ via IP₃—both of which are critical PKC activators [14]. Notably, this interaction appears to occur independently of the ion-regulatory function of Na+/K+-ATPase [39], although certain aspects of these pathways remain controversial [40]. Intriguingly, when OUA was combined with a PKCi, PKC activity did not increase, yet the reduction did not fall below control levels. This suggests that the respective effects of OUA and tamoxifen—the PKCi selected for this study—may have neutralized each other.

When combined with a PKCi, OUA failed to reduce Na+/K+-ATPase activity in both the in vitro and in vivo protocols. Furthermore, this combination prevented OUA from inducing the behavioral alterations characteristic of this animal model. These findings suggest that PKC may regulate Na+/K+-ATPase, and that both the OUA-induced inhibition of the enzyme and its subsequent behavioral outcomes could depend, at least in part, on PKC activation. This is consistent with a previous report from our research group [28]. Indeed, Feschenko and Sweadner [41] previously identified serine residues 11 and 18 within Na+/K+-ATPase as potential PKC phosphorylation sites. Bioinformatic structural analyses further support serine 11 and tyrosine 5 as key phosphorylation sites involved in enzyme regulation [42]. Additionally, the phosphorylation of serine 18 appears to play a central role in dopamine-induced Na+/K+-ATPase internalization [43]. Given these insights, we hypothesize that OUA-mediated inhibition of Na+/K+-ATPase activates PKC, which in turn phosphorylates Na+/K+-ATPase and triggers its internalization. Supporting this theory, Mohan et al. [44] reported that phosphorylation by PKC and PKG inhibits Na+/K+-ATPase activity, reinforcing the critical role of these kinases in enzyme regulation. However, a few limitations must be noted: (1) serine 18 is absent in several mammalian species, which may limit the generalizability of these findings [45]; and (2) OUA-induced internalization can occur independently of the Src pathway, indicating the involvement of alternative intracellular mechanisms [46].

It is important to emphasize that although tamoxifen is widely used in the biomedical literature and in mania models as a PKC inhibitor [21, 47, 48], it has pleiotropic effects and acts on other cellular targets, including estrogen receptors [49], directly modulating oxidative stress and calcium channels [36, 50]. Therefore, the reversal of ouabain-induced effects by tamoxifen observed in both in vitro and in vivo protocols suggests an intricate signaling network but precludes the assertion of direct and exclusive molecular crosstalk between PKC and Na⁺/K⁺-ATPase. The use of tamoxifen as a ‘PKC inhibitor’ in experimental models should be interpreted with caution, recognizing that its effects may result from multiple cellular actions. This lack of specificity limits the ability to attribute observed effects exclusively to PKC inhibition, necessitating more selective inhibitors or complementary genetic approaches to validate PKC’s specific role in the phenotypes studied. However, tamoxifen was strategically selected for the present study due to its high translational and clinical relevance. Since our investigation uniquely couples preclinical experiments (in vitro and in vivo) and a human clinical evaluation, utilizing tamoxifen—a drug widely investigated as a treatment for acute mania in BD—enabled us to establish a direct bridge between our animal findings and patient data, thereby strengthening the mechanistic interpretability and clinical applicability of our results.

Interestingly, the clinical study revealed that erythrocyte Na+/K+-ATPase activity was reduced, whereas erythrocyte PKC activity was increased in BD patients compared to healthy controls. Previous literature has underscored the role of Na+/K+-ATPase in BD pathogenesis, particularly the reduction in its activity, potentially driven by alterations in endogenous cardiac steroids, such as OUA [7, 12]. Additionally, PKC activity is known to be altered in both BD patients and various animal models of the disorder, a connection that underpins the potential use of tamoxifen as an alternative treatment [28, 51, 52]. However, in the present study, both the correlation and linear regression analyses between PKC and Na+/K+-ATPase were non-significant. A plausible explanation for this is that most participants were experiencing depressive or euthymic episodes, as reported by Valvassori and colleagues [8]. This clinical observation aligns with the lack of significant alterations in Na+/K+-ATPase and PKC activities 14 days post-OUA injection, which mimics a depressive-like state in animals. Taken together, these clinical findings corroborate the presence of a concomitant dysfunction in both the PKC and Na+/K+-ATPase pathways in BD. Rather than proposing a unidirectional, mechanistic regulation of one enzyme by the other, we argue that both pathways are integrated into a broader pathophysiological cascade involving cellular homeostasis and oxidative stress [53–55]. Within the framework of systems and network neurobiology, these systems do not function as isolated linear pathways, but rather as interconnected nodes of a complex cellular network. Consequently, the therapeutic effects of tamoxifen may be attributed to its ability to mitigate this systemic imbalance, restoring network stability rather than merely modulating a single target. Mounting evidence indicates that the inhibition of Na+/K+-ATPase by OUA generates severe oxidative and nitrosative stress [55–57]. This oxidative environment, in turn, acts as a potent activator of various PKC isoforms, which can further exacerbate intracellular ROS/RNS production [58, 59]. Consequently, the protective effects of tamoxifen observed in this study may occur by disrupting this vicious cycle. Rather than implying a direct, physical protein-protein interaction, we argue that tamoxifen stabilizes Na+/K+-ATPase activity indirectly. By mitigating the noxious oxidative microenvironment that would otherwise degrade or inhibit the sodium pump, tamoxifen breaks the feedback loop, thereby preserving enzyme integrity and cellular homeostasis.

To understand the translational alignment between our preclinical models and clinical findings, the fundamental differences between an acute pharmacological challenge and chronic human BD must be considered. In the rat model, ICV ouabain causes acute enzyme disruption that normalizes by day 14, leaving secondary downstream cascades (such as persistent oxidative stress) to sustain depressive-like behaviors [27]. In contrast, human BD is a chronic systemic condition, and evidence suggests that reduced erythrocyte Na+/K+-ATPase activity and elevated PKC signaling serve as persistent trait markers of cellular membrane dysregulation, remaining altered even during depressive or euthymic states [14, 60]. Although brain tissue and blood cells perform different roles, peripheral erythrocytes and leukocytes share essential cellular machinery with neurons, including membrane transport systems and intracellular kinase signaling cascades. Because of this shared machinery, blood cells serve as valuable and accessible representatives for the systemic cellular pathology underlying BD.

Indeed, in the present study, OUA increased the levels of oxidative and nitrosative damage markers—specifically 4-HNE, MDA, 8-ISO, carbonyl, and 3-nitrotyrosine—in both the in vivo and in vitro protocols. All of these alterations were reversed by PKCi, except for 8-ISO in the in vitro protocol. OUA is well known to induce oxidative and nitrosative stress across various experimental settings [55, 56]. Some studies suggest that these OUA-induced oxidative and nitrosative injuries stem from disruptions in Na + and K+ homeostasis [27]. Furthermore, Zhang et al. [14] indicated that ROS can induce Na+/K+-ATPase endocytosis, thereby reducing its membrane density. Conversely, OUA-induced impairment of Na+/K+-ATPase activity can trigger ROS production via the Ras/Raf/MEK/ERK1/2 pathway, thereby establishing a pathological positive feedback loop. In the clinical arm of this study, linear regression analysis identified Na+/K+-ATPase as a statistical predictor of serum 4-HNE and 8-ISO levels in BD patients. Additionally, correlation analysis revealed a negative correlation between Na+/K+-ATPase activity and carbonyl levels. Taken together, these results reinforce the existence of a positive feedback loop between reduced Na+/K+-ATPase activity and elevated oxidative stress, both of which are critical components of BD pathophysiology [4, 10, 26]. However, these analyses yielded weak to very weak coefficients, suggesting that other factors also influence the interplay between Na+/K+-ATPase and oxidative stress.

Herein, we also demonstrate PKC-dependent oxidation and nitration of lipids and proteins in brain cells induced by OUA. This indicates that the positive feedback loop between ROS/RNS and Na+/K+-ATPase could, at least in part, depend on PKC activity. Indeed, the role of PKC in oxidative stress is already established by previous studies [22], where a PKCi reversed and prevented amphetamine-induced oxidative stress in an animal model of mania. Further investigations found that the PKCε isoform was the most closely associated with the oxidative stress and behavioral alterations induced by this dopaminergic animal model [28]. Considering that the PKCε isoform belongs to the novel PKC family [15], its activation is primarily correlated with DAG production rather than calcium levels. However, further studies are required to fully elucidate the mechanism underlying the correlation between the PKCε isoform and the production of oxidative stress.

An intriguing finding in this study is the temporal uncoupling observed on day 14, in which depressive-like behavioral phenotypes—indicated by decreased grooming in the splash test—are observed despite normalization of both Na+/K+-ATPase and PKC activities. Regarding the pharmacokinetics of these compounds, ouabain binds with high affinity to the alpha-subunit of Na+/K+-ATPase [12], sustaining enzyme inhibition and downstream signaling throughout the first week post-infusion. Over time, endogenous cellular mechanisms, including pump endocytosis, lysosomal degradation, and de novo protein synthesis, gradually restore baseline enzymatic function [46, 61, 62], leading to the normalization of both Na+/K+-ATPase (27) and PKC activities by day 14. Meanwhile, tamoxifen rapidly penetrates brain tissue upon intracerebroventricular delivery, acting within the critical acute window to block PKC hyperactivation and downstream oxidative damage [20, 22, 48]. This discrepancy suggests that the acute and severe disruption of cellular homeostasis at day 7, characterized by peak oxidative stress and concurrent enzymatic alterations, triggers a secondary cascade of downstream pathological processes—such as neuroinflammation, reductions in neurotrophic factors, and persistent macromolecular damage—that endure beyond the biochemical recovery of the enzymes [12, 37, 55, 60] to sustain depressive-like behavior at day 14 [27].

**Fig. 7:**
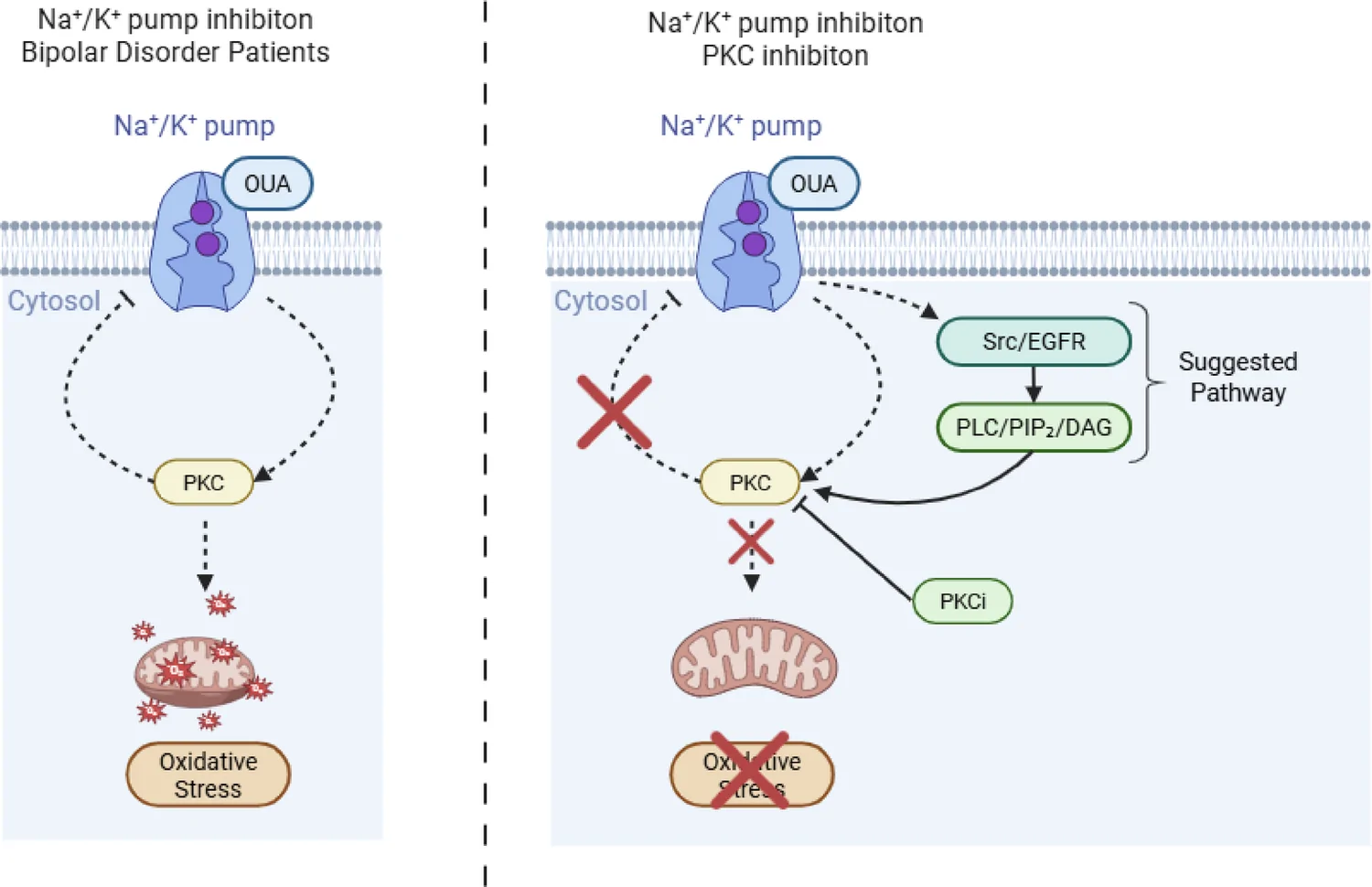
Visual representation on the interaction between Na^+^/K^+^ pump and PKC in the context of bipolar disorder. OUA ouabain, PKC protein kinase C, PKCi PKC inhibitor, EGFR epidermal growth factor receptor, PLC phospholipase C, PIP_2_ phosphatidylinositol 4,5-bisphosphate, DAG diacylglycerol. Created in BioRender. Valvassori, S. (2026) https://BioRender.com/vx9jquo

Taken together, these translational findings—spanning in vitro, preclinical in vivo, and clinical data—indicate that Na+/K+-ATPase and PKC are critical components of BD pathophysiology. These insights open new avenues for developing novel theoretical frameworks and therapeutic strategies for BD. However, current literature still exhibits gaps regarding the precise relationship between these two systems, particularly within central nervous system tissues. Therefore, further studies are warranted to fully elucidate this molecular crosstalk directly in brain tissue. A visual representation of the model proposed can be found in Fig. 7.

## Limitations and Future Research Directions

Some important limitations should be pointed out: (1) All the analyses were conducted using the whole brain, which could lead to a non-specific result; (2) It is important to point out that the preclinical biochemical analyses were performed in brain tissue and the clinical analyses were performed on serum/plasma samples. Both samples present different metabolic characteristics, which lead to significant differences between the results of oxidative stress markers; (3) For some parameters, such as visits to the center of the open field, there was some variability in the groups, which could have limited the results of this study.

Considering the results found in the present research, further studies should focus on the analysis of different subunits of PKC and the different brain regions linked to BD pathophysiology. Additionally, different behavioral analyses, such as the forced swim test and the tail suspension test, could enrich the relationship between the effects of PKC and the alterations caused by OUA.

We formally acknowledge the lack of direct molecular evidence to definitively establish causal crosstalk between PKC and Na+/K+-ATPase, rather than parallel, independent signaling events. Specifically, future studies should evaluate the post-translational phosphorylation of the Na+/K+-ATPase α-subunit (e.g., at Ser-18 or Ser-23) and track its endocytic internalization and plasma membrane availability. Furthermore, while the use of tamoxifen is clinically relevant, it introduces potential non-specific, pleiotropic effects. To strengthen these mechanistic conclusions, subsequent investigations should employ highly selective pharmacological tools—such as Ro-31–8220, GF109203X, or chelerethrine—as well as peptide inhibitors and genetic approaches, including RNA interference or isoform-specific PKC knockout mice. These tools will be fundamental to unravel the exact molecular pathways, to determine whether PKC activation is driven by phosphorylation of specific residues or by changes in overall expression and characterize the signaling cascades responsible for the dynamic recovery of enzyme activity observed on day 14. Despite these mechanistic limitations, the present study provides a valuable translational contribution. By bridging preclinical models and human clinical data, this work demonstrates that a pharmacological intervention targeting these interconnected pathways effectively mitigates the combined biochemical, oxidative, and behavioral dysfunctions that closely mirror the complex pathophysiology of BD.

Additionally, the preclinical in vivo experiments were conducted exclusively in male rats to avoid estrous cycle variability, whereas the clinical sample was predominantly female. Given that tamoxifen possesses pleiotropic actions, including selective estrogen receptor modulation, potential sex-specific effects on PKC signaling, Na+/K+-ATPase regulation, and behavior cannot be excluded and warrant systematic evaluation in female animal models across different estrous phases in future studies.

Furthermore, our clinical study was predominantly composed of patients in depressive (*n* = 23) or euthymic (*n* = 7) phases, with a small representation of manic (*n* = 3) and mixed (*n* = 3) states. Consequently, the study was underpowered to evaluate state-dependent differences in Na+/K+-ATPase and PKC activities across distinct mood episodes, warranting larger, state-stratified clinical cohorts in future research.

## Data Availability

The datasets generated during and/or analyzed during the current study are not publicly available but are available from the corresponding author on reasonable request.

## References

[1] Clemente AS, Diniz BS, Nicolato R et al (2015) Bipolar disorder prevalence: a systematic review and meta-analysis of the literature. Braz J Psychiatry 37:155–161. 10.1590/1516-4446-2012-1693 25946396

[2] Madireddy S, Madireddy S (2022) Therapeutic interventions to mitigate mitochondrial dysfunction and oxidative stress-induced damage in patients with bipolar disorder. Int J Mol Sci. 10.3390/IJMS23031844 PMC8836876 35163764

[3] McIntyre RS, Berk M, Brietzke E et al (2020) Bipolar disorders. Lancet 396:1841–1856. 10.1016/S0140-6736(20)31544-0 33278937

[4] Lichtstein D, Ilani A, Rosen H et al (2018) Na+, K+-ATPase signaling and bipolar disorder. Int J Mol Sci 19:2314. 10.3390/IJMS19082314 PMC6121236 30087257

[5] Clausen MV, Hilbers F, Poulsen H (2017) The structure and function of the Na,K-ATPase isoforms in health and disease. Front Physiol 8:257141. 10.3389/FPHYS.2017.00371/BIBTEX PMC5459889 28634454

[6] El-Mallakh RS (1983) The Na,K-ATPase hypothesis for manic-depression. I. General considerations. Med Hypotheses 12:253–268. 10.1016/0306-9877(83)90042-7 6321918

[7] El-Mallakh RS, Wyatt RJ (1995) The Na,K-ATPase hypothesis for bipolar illness. Biol Psychiatry 37:235–244. 10.1016/0006-3223(94)00201-D 7711160

[8] Valvassori SS, Peper-Nascimento J, Aguiar-Geraldo JM et al (2023) Biological rhythms are correlated with Na+, K+-ATPase and oxidative stress biomarkers: A translational study on bipolar disorder. J Affect Disord 340:877–885. 10.1016/j.jad.2023.08.042 37572705

[9] Hodes A, Rosen H, Deutsch J et al (2016) Endogenous cardiac steroids in animal models of mania. Bipolar Disord 18:451–459. 10.1111/BDI.12413 27393337

[10] Horesh N, Pelov I, Pogodin I et al (2024) Involvement of the Na+, K+-ATPase α1 isoform and endogenous cardiac steroids in depression- and manic-like behaviors. Int J Mol Sci. 10.3390/IJMS25031644 PMC10855204 38338921

[11] Goldstein I, Levy T, Galili D et al (2006) Involvement of Na(+), K(+)-ATPase and endogenous digitalis-like compounds in depressive disorders. Biol Psychiatry 60:491–499. 10.1016/J.BIOPSYCH.2005.12.021 16712803

[12] El-Mallakh RS, Sampath VP, Horesh N, Lichtstein D (2022) Endogenous cardiac steroids in bipolar disorder: state of the art. Int J Mol Sci. 10.3390/IJMS23031846 PMC8836531 35163766

[13] Grider G, El-Mallakh RS, O’Malley Huff M et al (1999) Endogenous digoxin-like immunoreactive factor (DLIF) serum concentrations are decreased in manic bipolar patients compared to normal controls. J Affect Disord 54:261–267. 10.1016/S0165-0327(98)00208-0 10467969

[14] Zhang L, Zhang Z, Guo H, Wang Y (2008) Na+/K+-ATPase-mediated signal transduction and Na+/K+-ATPase regulation. Fundam Clin Pharmacol 22:615–621. 10.1111/J.1472-8206.2008.00620.X 19049666

[15] Newton AC (2018) Protein kinase C: perfectly balanced. Crit Rev Biochem Mol Biol 53:208. 10.1080/10409238.2018.1442408 PMC5901981 29513138

[16] Zeng L, Webster SV, Newton PM (2012) The biology of protein kinase C. Adv Exp Med Biol 740:639–661. 10.1007/978-94-007-2888-2_28 22453963

[17] Yuan Z, Cai T, Tian J et al (2005) Na/K-ATPase tethers phospholipase C and IP3 receptor into a calcium-regulatory complex. Mol Biol Cell 16:4034–4045. 10.1091/MBC.E05-04-0295 PMC1196317 15975899

[18] Bertorello AM, Ridge KM, Chibalin AV et al (1999) Isoproterenol increases Na+-K+-ATPase activity by membrane insertion of α-subunits in lung alveolar cells. Am J Physiol Lung Cell Mol Physiol 276:L20–L27 10.1152/ajplung.1999.276.1.L20 9887051

[19] Dada LA, Sznajder JI (2003) Mechanisms of pulmonary edema clearance during acute hypoxemic respiratory failure: role of the Na,K-ATPase. Crit Care Med. 10.1097/01.CCM.0000057895.22008.EC. 31:12682448

[20] Dal-Pont GC, Resende WR, Bianchini G et al (2019) Tamoxifen has an anti-manic effect but not protect the brain against oxidative stress in an animal model of mania induced by ouabain. J Psychiatr Res 113:181–189. 10.1016/J.JPSYCHIRES.2019.03.020 30981159

[21] Valvassori SS, Bavaresco DV, Budni J et al (2014) Effects of tamoxifen on tricarboxylic acid cycle enzymes in the brain of rats submitted to an animal model of mania induced by amphetamine. Psychiatry Res 215:483–487. 10.1016/j.psychres.2013.11.016 24359811

[22] Valnier Steckert A, Silva Valvassori S, Mina F et al (2012) Protein kinase C and oxidative stress in an animal model of mania. Curr Neurovasc Res 9:47–57. 10.2174/156720212799297056 22272765

[23] Palacios J, Yildiz A, Young AH, Taylor MJ (2019) Tamoxifen for bipolar disorder: systematic review and meta-analysis. J Psychopharmacol 33:177–184. 10.1177/0269881118822167 30741085

[24] Sies H (2015) Oxidative stress: a concept in redox biology and medicine. Redox Biol 4:180–183. 10.1016/J.REDOX.2015.01.002 PMC4309861 25588755

[25] Klandorf H, van Dyke K, Klandorf H, van Dyke K (2012) Oxidative and nitrosative stresses: their role in health and disease in man and birds. Oxidat Stress Mol Mech Biol Effects. 10.5772/33879

[26] Kato T (2019) Current understanding of bipolar disorder: Toward integration of biological basis and treatment strategies. Psychiatry Clin Neurosci 73:526–540. 10.1111/PCN.12852 31021488

[27] Valvassori SS, Dal-Pont GC, Resende WR et al (2019) Validation of the animal model of bipolar disorder induced by ouabain: face, construct and predictive perspectives. Transl Psychiatry. 10.1038/s41398-019-0494-6 PMC6548776 31164628

[28] Valvassori S, Cararo SH, Peper-Nascimento J J, et al (2020) Protein kinase C isoforms as a target for manic-like behaviors and oxidative stress in a dopaminergic animal model of mania. Prog Neuropsychopharmacol Biol Psychiatry. 10.1016/j.pnpbp.2020.109940. 101:32243997

[29] Nabha SM, Glaros S, Hong M et al (2005) Upregulation of PKC-delta contributes to antiestrogen resistance in mammary tumor cells. Oncogene 24:3166–3176. 10.1038/SJ.ONC.1208502 15735693

[30] Paxinos George W (2013) Charles the rat brain in stereotaxic coordinates: Hard Cover Edition. 466

[31] Taksande BG, Faldu DS, Dixit MP et al (2013) Agmatine attenuates chronic unpredictable mild stress induced behavioral alteration in mice. Eur J Pharmacol 720:115–120. 10.1016/J.EJPHAR.2013.10.041 24183973

[32] Wyse ATS, Streck EL, Barros SVT et al (2000) Methylmalonate administration decreases Na+,K+-ATPase activity in cerebral cortex of rats. NeuroReport 11:2331–2334. 10.1097/00001756-200007140-00052 10923695

[33] Chan KM, Delfert D, Junger KD (1986) A direct colorimetric assay for Ca2+ -stimulated ATPase activity. Anal Biochem 157:375–380. 10.1016/0003-2697(86)90640-8 2946250

[34] Kimura M, Yokoyama A, Higuchi S (2019) Aldehyde dehydrogenase-2 as a therapeutic target. Expert Opin Ther Targets 23:955–966. 10.1080/14728222.2019.1690454 31697578

[35] American Psychiatric Association (2013) Diagnostic and statistical manual of mental disorders. Diagnostic and statistical manual of mental disorders. 10.1176/APPI.BOOKS.9780890425596

[36] Ahmed NS, Samec M, Liskova A et al (2021) Tamoxifen and oxidative stress: an overlooked connection. Discov Oncol. 10.1007/S12672-021-00411-Y PMC8777555 35201439

[37] Possamai-Della T, Dal-Pont GC, Resende WR et al (2022) Imipramine can be effective on depressive-like behaviors, but not on neurotrophic factor levels in an animal model for bipolar disorder induced by ouabain. Mol Neurobiol. 10.1007/s12035-022-03022-y 36121567

[38] Cui X, Xie Z (2017) Protein interaction and Na/K-ATPase-mediated signal transduction. Molecules. 10.3390/MOLECULES22060990 PMC6152704 28613263

[39] Aperia A (2007) New roles for an old enzyme: Na,K-ATPase emerges as an interesting drug target. J Intern Med 261:44–52. 10.1111/J.1365-2796.2006.01745.X 17222167

[40] Askari A (2019) The other functions of the sodium pump. Cell Calcium 84:102105. 10.1016/J.CECA.2019.102105 31733624

[41] Feschenko MS, Sweadner KJ (1997) Phosphorylation of Na,K-ATPase by protein kinase C at Ser18 occurs in intact cells but does not result in direct inhibition of ATP hydrolysis. J Biol Chem 272:17726–17733. 10.1074/JBC.272.28.17726 9211924

[42] Blayney EL, Chennath M, Cranfield CG, Clarke RJ (2023) Bioinformatic analysis of Na+, K+-ATPase regulation through phosphorylation of the alpha-subunit N-terminus. Int J Mol Sci. 10.3390/IJMS24010067 PMC9820343 36613508

[43] Chibalin AV, Ogimoto G, Pedemonte CH et al (1999) Dopamine-induced endocytosis of Na+,K+-ATPase is initiated by phosphorylation of Ser-18 in the rat α subunit and is responsible for the decreased activity in epithelial cells. J Biol Chem 274:1920–1927. 10.1074/JBC.274.4.1920 9890946

[44] Mohan S, Tiwari MN, Biala Y, Yaari Y (2019) Regulation of neuronal Na+/K+-ATPase by specific protein kinases and protein phosphatases. J Neurosci 39:5440. 10.1523/JNEUROSCI.0265-19.2019 PMC6616281 31085608

[45] Hodeify R, Kreydiyyeh S, Zaid LMJ (2023) Identified and potential internalization signals involved in trafficking and regulation of Na+/K+ ATPase activity. Mol Cell Biochem 479(7):1583–1598. 10.1007/S11010-023-04831-Y PMC11254989 37634170

[46] Cherniavsky-Lev M, Golani O, Karlish SJD, Garty H (2014) Ouabain-induced internalization and lysosomal degradation of the Na+/K+-ATPase. J Biol Chem 289:1049–1059. 10.1074/JBC.M113.517003 PMC3887173 24275648

[47] Valvassori SS, Borges CP, Varela RB et al (2017) The different effects of lithium and tamoxifen on memory formation and the levels of neurotrophic factors in the brain of male and female rats. Brain Res Bull 134:228–235. 10.1016/j.brainresbull.2017.08.006 28802897

[48] Valvassori SS, Dal-Pont GC, Resende WR et al (2017) Lithium and tamoxifen modulate behavior and protein kinase C activity in the animal model of mania induced by ouabain. Int J Neuropsychopharmacol 20:877–885. 10.1093/IJNP/PYX049 PMC5737643 29020306

[49] Khan MM (2018) Translational significance of selective estrogen receptor modulators in psychiatric disorders. Int J Endocrinol. 10.1155/2018/9516592 PMC6196929 30402099

[50] Radin DP, Patel P (2016) Delineating the molecular mechanisms of tamoxifen’s oncolytic actions in estrogen receptor-negative cancers. Eur J Pharmacol 781:173–180. 10.1016/j.ejphar.2016.04.017 27083550

[51] Machado-Vieira R, Courtes AC, Zarate CA et al (2023) Non-canonical pathways in the pathophysiology and therapeutics of bipolar disorder. Front Neurosci. 10.3389/FNINS.2023.1228455 PMC10427509 37592949

[52] Bagdadi N, Azab AN, Shvartsur R (2021) The use of tamoxifen as a potential treatment for bipolar disorder. Psychiatry Clin Psychopharmacol 31:344–352. 10.5152/PCP.2021.21817 PMC11079652 38765942

[53] Teleanu DM, Niculescu AG, Lungu II et al (2022) An overview of oxidative stress, neuroinflammation, and neurodegenerative diseases. Int J Mol Sci. 10.3390/IJMS23115938 PMC9180653 35682615

[54] Gui Z, Zhang Q, Sha S et al (2024) Bipolar disorder and oxidative stress: a bibliometric perspective. Asia Pac Psychiatry. 10.1111/APPY.12564 39136098

[55] Riegel RE, Valvassori SS, Moretti M et al (2010) Intracerebroventricular ouabain administration induces oxidative stress in the rat brain. Int J Dev Neurosci 28:233–237. 10.1016/J.IJDEVNEU.2010.02.002 20153819

[56] Aguiar-Geraldo JM, Possamai-Della T, Menegas S et al (2023) Folic acid does not have an anti-manic effect but protect the brain against oxidative stress in an animal model of mania induced by ouabain. J Affect Disord. 10.1016/j.jad.2023.04.137 PMC10464577 37150224

[57] Jornada LK, Valvassori SS, Steckert AV et al (2011) Lithium and valproate modulate antioxidant enzymes and prevent ouabain-induced oxidative damage in an animal model of mania. J Psychiatr Res 45:162–168. 10.1016/J.JPSYCHIRES.2010.05.011 20627318

[58] Numazawa S, Ishikawa M, Yoshida A et al (2003) Atypical protein kinase C mediates activation of NF-E2-related factor 2 in response to oxidative stress. Am J Physiol Cell Physiol. 10.1152/AJPCELL.00043.2003 12700136

[59] Fei YD, Li W, Hou JW et al (2017) Oxidative stress-induced afterdepolarizations and protein kinase C signaling. Int J Mol Sci. 10.3390/IJMS18040688 PMC5412274 28358314

[60] El-Mallakh RS, Gao Y, You P (2021) Role of endogenous ouabain in the etiology of bipolar disorder. Int J Bipolar Disord 9:6. 10.1186/S40345-020-00213-1 PMC7851255 33523310

[61] Klimanova EA, Tverskoi AM, Koltsova SV et al (2017) Time- and dose dependent actions of cardiotonic steroids on transcriptome and intracellular content of Na + and K +: A comparative analysis. Sci Rep 7:45403–. 10.1038/SREP45403;TECHMETA PMC5366943 28345607

[62] Schmidt BE, Leite AKO, Farias CP et al (2026) Regional crosstalk between the amygdala, hippocampus, and prefrontal cortex following Na+,K+-ATPase inhibition by ouabain. Neurotox Res. 10.1007/S12640-026-00800-7 PMC13152888 42096113

